# Developing an SDN security model (EnsureS) based on lightweight service path validation with batch hashing and tag verification

**DOI:** 10.1038/s41598-023-44701-7

**Published:** 2023-10-13

**Authors:** S. Pradeep, Yogesh Kumar Sharma, Umesh Kumar Lilhore, Sarita Simaiya, Abhishek Kumar, Sachin Ahuja, Martin Margala, Prasun Chakrabarti, Tulika Chakrabarti

**Affiliations:** 1 Department of Computer Science and Engineering, Malla Reddy Engineering College for Women, UGC Autonomous Institution, Maisammaguda, Secunderabad, Telangana India; 2 Department of Computer Science and Engineering, Koneru Lakshmaiah Education Foundation, Vaddeswaram, Guntur, AP India; 3 Department of Computer Science and Engineering, Chandigarh University, Gharuan, Mohali, Punjab 140413 India; 4 APEX Institute of Technology (AIT), CSE, Chandigarh University, Gharuan, Mohali, Punjab 140413 India; 5 Department of Computer Science and Engineering, Chandigarh University, Gharuan, Mohali, Punjab 140413 India; 6 School of Computing, University of Louisiana at Lafayette, Lafayette, USA; 7 Sir Padampat, Singhania University, Udaipur, Rajasthan 313601 India

**Keywords:** Electrical and electronic engineering, Energy science and technology, Engineering, Mathematics and computing

## Abstract

Software-defined networking (SDN) has significantly transformed the field of network management through the consolidation of control and provision of enhanced adaptability. However, this paradigm shift has concurrently presented novel security concerns. The preservation of service path integrity holds significant importance within SDN environments due to the potential for malevolent entities to exploit network flows, resulting in a range of security breaches. This research paper introduces a model called "EnsureS", which aims to enhance the security of SDN by proposing an efficient and secure service path validation approach. The proposed approach utilizes a Lightweight Service Path Validation using Batch Hashing and Tag Verification, focusing on improving service path validation's efficiency and security in SDN environments. The proposed EnsureS system utilizes two primary techniques in order to validate service pathways efficiently. Firstly, the method utilizes batch hashing in order to minimize computational overhead. The proposed EnsureS algorithm enhances performance by aggregating packets through batches rather than independently; the hashing process takes place on each one in the service pathway. Additionally, the implementation of tag verification enables network devices to efficiently verify the authenticity of packets by leveraging pre-established trust relationships. EnsureS provides a streamlined and effective approach for validating service paths in SDN environments by integrating these methodologies. In order to assess the efficacy of the Proposed EnsureS, a comprehensive series of investigations were conducted within a simulated SDN circumstance. The efficacy of Proposed EnsureS was then compared to that of established methods. The findings of our study indicate that the proposed EnsureS solution effectively minimizes computational overhead without compromising on the established security standards. The implementation successfully reduces the impact of different types of attacks, such as route alteration and packet spoofing, increasing SDN networks' general integrity.

## Introduction

A well-known concept called SFC enables the provision of numerous network services under varied restrictions based on the needs of individual applications. For instance, telemedicine and tele-surgery applications require security services with low-latency connectivity^1^. Some applications also have special requirements, such as dedicated bandwidth for various applications, i.e., virtual machines (VMs) migration, low latency for financial transactions, low priority for online gaming, etc. To use these services, the application track must pass through Network Functions (NFs) in a specific order, such as Firewall-IDS-NAT, while under time restrictions. Due to the introduction of new applications, SFC is becoming increasingly in demand^2^.

Emerging technologies like 5G, edge computing, the Internet of Things, etc., aspire to install flexible and dynamic SFCs to meet the varied demands of applications. NFV and SDN are two such technologies. Although it has many benefits, the SDN/NFV ecosystem presents several security risks because of software flaws^3^. In the case of SFC, these flaws can be utilized to attack a particular NF and network service. For instance, an extreme threat in SFC is NF bypassing, where the security services can be bypassed by compromising the software switches^4^.

**Figure 1:**
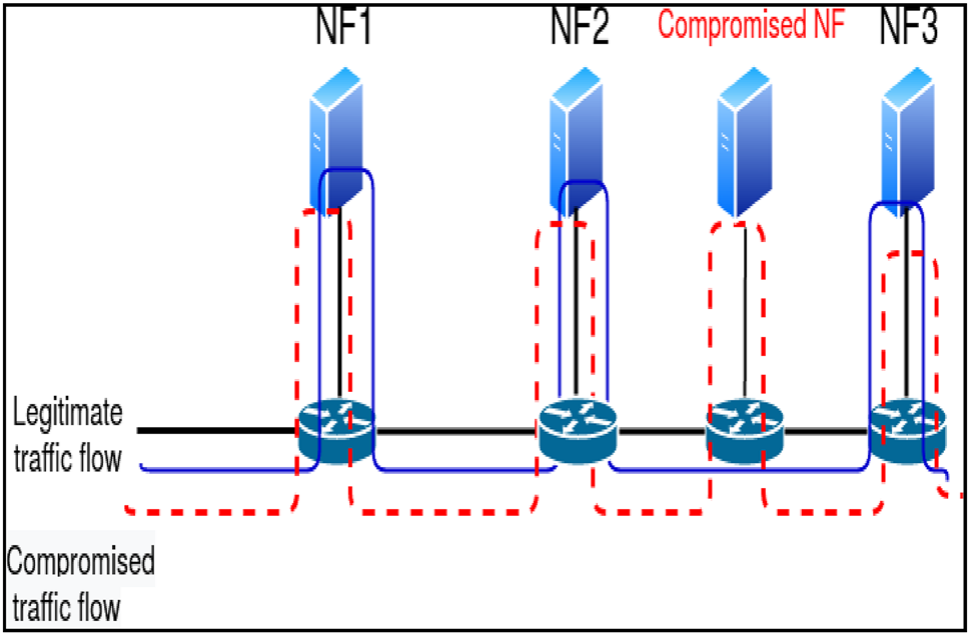
Network function by passing attack by compromised switch.

Another danger to Service Function Chaining (SFC) is packet manipulation, where attackers can use the packet modification capabilities of software switches and VNFs to tamper with packets or introduce malicious code. Yet, many NFs that are a part of SFC also carry out simple packet modification. Consequently, in the case of SFC, it is vital to identify and avoid undesired packet alteration. By verifying the path a packet takes, service function path validation is a viable method of detecting various security concerns. The current hop-by-hop tag verification^5^ approaches protect against packet modification attacks and ensure path integrity but come with a hefty processing cost per NF because of encryption-decryption operations. Hop-by-hop encryption-decryption also causes packet forwarding to lag, limiting this technique's use for low-latency applications. As Fig. 1 presents, the NF attack occurs when a compromised switch does not forward packets to its attached middlebox.

### (A) Security attacks in SFC

The switches and links linking controls or NFs may be affected due to software flaws and setup errors. A hypervisor of VNFs in NFV can also be compromised^6^ because of a software flaw or unapproved access. This section demonstrates security attacks that could be launched on SFC using these flaws. This part also describes a brand-new security risk called an NF misleading attack. NF bypassing attack: The hacked switches can reroute traffic to the next switch or NF, as illustrated in Fig. 1 by the red dotted line, and purposefully fail to send traffic to the necessary NF.

- Out-of-order traversal: compromised switches can reorder the traffic flow.
- The attack involving packet tampering: a hacker uses switches' or VNFs' ability to modify packets to tamper with them or introduce harmful data.

There are various methods for carrying out packet manipulation^7^.

**Figure 2:**
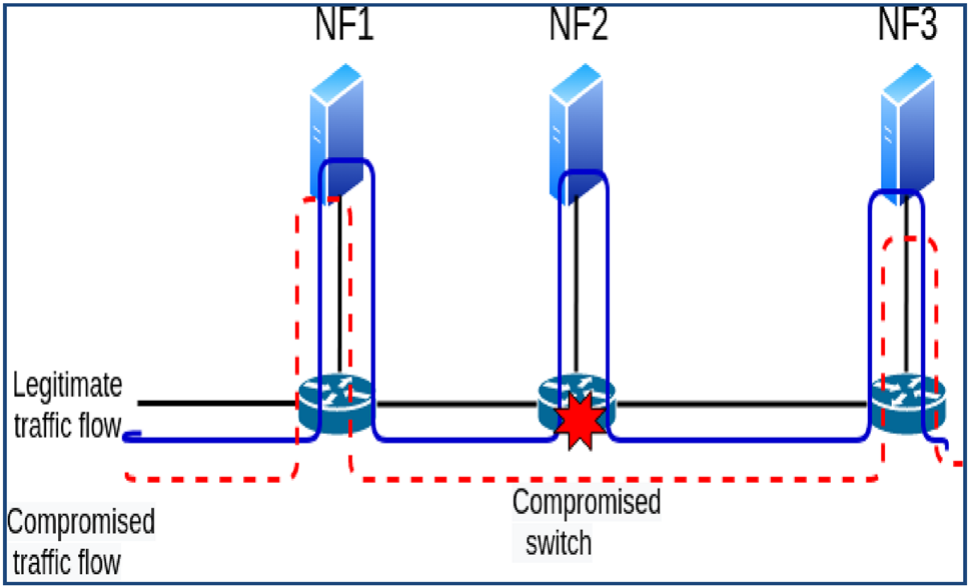
Packet modification by compromised link.

**Figure 3:**
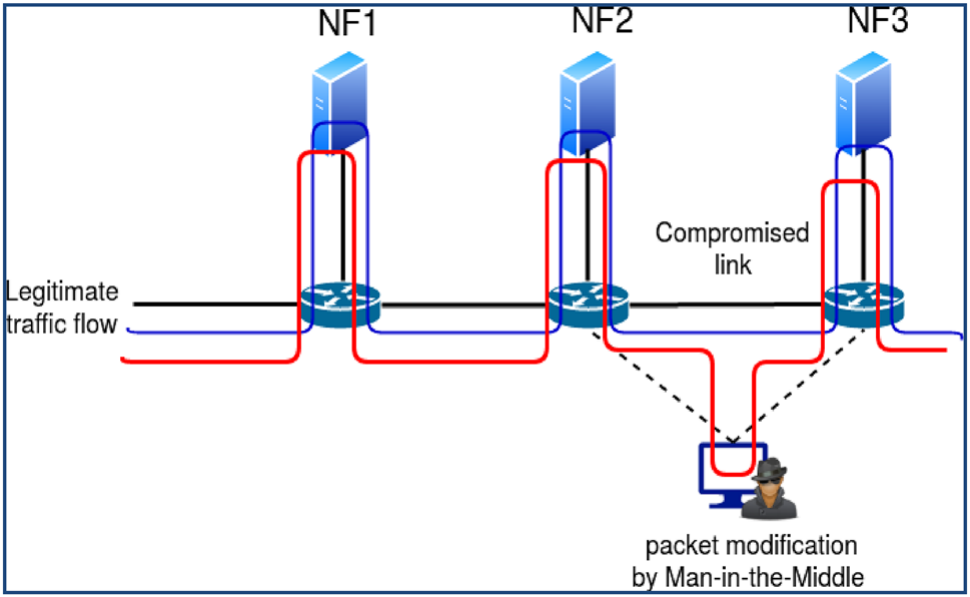
Packet modification by compromised VNF.

**Figure 4:**
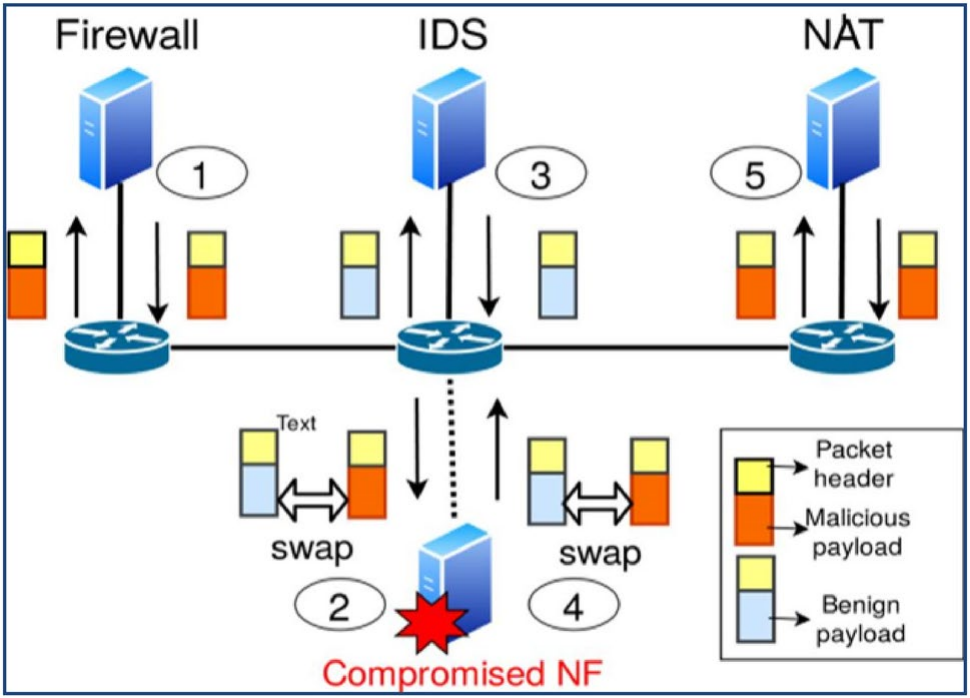
Network function misleading attack.

- Insecure switch: attackers can alter packets by changing the header and payload using software switches' packet modification capabilities.
- Actively changing the link as a man-in-the-middle: using a link fabrication attack, an attacker can compromise the link between switches or the link linking NF, as demonstrated in Fig. 2. After IDS/IPS has processed the packet, an attacker utilizes a compromised link to actively modify the traffic by introducing malicious data, for example.
- VNF that has been compromised: the VNF may have been affected by the hypervisor's weakness, which was utilized to install the VNF^8^. A hacked VNF of this nature can actively carry out packet modification in Fig. 3.
- NF misleading attack: The packet can be used by a switch or VNF that has been compromised, modifying capabilities to trick the security services and let outside malicious traffic into the network^9^. The misleading attack by the NF is depicted in Fig. 4. Please assume that the inbound packet's payload contains negative data, forcing it to pass via the SFC: FW-IDS-NAT.

The attacker wants to trick the IDS into allowing the packet network even though, in an ideal world, the IDS would reject the packet^10^. According to the SFC, the layer-3 firewall (FW) first processes the malicious packet. The attacker then replaces the harmful payload with a benign one and sends the altered packet to the IDS. IDS do not notice the alteration because the packet is genuine and has no harmful material; therefore, it gets forwarded. The attacker now switches the packet's payload and transmits it to the NAT.

After NAT has processed the packet, malicious traffic can enter the network. The tag-based verification^11^ approach, where the packet header information is predominantly employed for tag generation and validation, cannot detect the NF deceptive attack by payload swapping. Table 1 presents the differentiation of detection potentialities of SFC validation outcomes.

**Table 1:** Differentiation of detection potentialities of SFC validation outcomes.

Method/	NF	Packet	NF
Attack	Bypassing	Tampering	Misleading
Flow-clock	✓	×	×
Probe-based	✓	✓	×
Footprint	✓	✓	×

### (B) Key contribution of the work

The key contribution of the work is as follows.

i. This research proposed an effective service path validation mechanism to lessen these risks. The proposed EnsureS model is based on Lightweight service path validation with batch hashing and tag verification to combat security threats.
ii. This research also describes the "NF-misleading attack", a new security risk for SFC. The attacker alters the packet payload in this threat to get the NF to approve the malicious traffic and also provides the solution by the proposed security model.
iii. We identify security weaknesses and rectify the reported security risks to develop a secure service path for SDN with the least resource consumption. In the proposed model, every NF verifies the packet hash to prevent packet alteration attacks.
iv. In experimental analysis, it is proven that compared to the existing validation method, the proposed model avoids packet modification attacks, significantly lowering the processing overhead per packet.

### (C) Organization of the article

The complete article is organized as follows: section two covers related work, section three covers materials and methods, section four covers results and discussion and section five covers the conclusion and future direction of the research.

## Related work

There have been several proposals for reducing security assaults in SFC based on probes, flow statistics, hop-by-hop tag verification, etc. The probe-based process^12^ injects probe packets into networks and verifies their integrity to validate the service path. Instead, the flow-statistic-based technique gathers and compares all the statistics from network components to identify statistical inconsistencies. Yet, these answers show that Switches can avoid probe- or statistics-based detection during an NF bypassing assault since they do not exhibit flow statistics volatility between switches^13^.

A hacked switch engages in harmful behaviour on active flow packets while excluding the probe packets. Flow-statistics-based validation is a coarse-grained method of identifying switch-forwarding patterns and adding extra overhead for statistics gathering. Numerous hop-by-hop tag verification techniques have recently been developed to counter the middlebox bypass attack^14^.

To get beyond the drawbacks of the aforementioned current approaches, the inventor of the Flow cloak took into account the middle box-bypass attack and designed a real-time and per-packet validation mechanism. This method uses the pre-shared deterministic tag and packet header information to generate and verify the tag at each NF. Yet, these tag-based validation methods continue to have security issues and add a lot of processing overhead^15^.

The ordered multi-signature approach for SFC validation Footprint^16^ is susceptible to similar attacks. The middleboxes belonging to the SFC must insert their signatures according to the defined order when using the Footprint method. In this approach, the first middlebox, FW, creates the signature by hashing the malicious packet before sending it to the second middlebox, IDS. The attacker switches the payload before sending the malicious packet to the IDS and then sends the altered packet with an FW signature.

An ID examines the valid package in this case and inserts its signature. The attacker swaps the payload again for the earlier malicious data and sends the packet to the NAT. Moreover, NAT added its signature and sent the packet to the verifier. The verifier function may correctly recreate the multi-signature by hashing the malicious packet^17^.

A few techniques, such as SPV, considered the threat of packet alteration and advocated hop-by-hop packet encryption decryption to counter the attack and maintain path integrity^18^. A Hop-by-hop-based encryption decryption security implementation can also cause a significant packet forwarding delay, which limits the use of this approach for low-latency applications^19^. By combining the control center for non-homogenous connected devices, an SDN may improve network setup and administration^20^. As a result, the entire system can be established with configurable devices and automatically optimized according to the overall network condition.

A Network administrator also enables the standardization of the control system with the global development of the underlying network, and it's anticipated to enhance system performance with the best possible use of the runtime environment. Besides that, SDN promotes the development of next-generation connections by providing an easy platform for evaluating new methodologies^21^.

## Materials and methods

This section covers the materials and methods used for the research.

### (A) Proposed ensures model

This research presents an Efficient SFC path validation scheme (EnsureS). The proposed SFC validation scheme and batch hashing enabling lightweight adoption of the proposed validation scheme are described in depth in this section^22^. The details of the proposed EnsureS are as follows.

### (B) Architecture and working of the proposed EnsureS model

The service route validation technique should ensure that both the path and the contents carried by the packet adhere to the NFs' predefined order of precedence. Such path validation can prevent packet manipulation attacks and provide path integrity.

We suggest EnsureS, an effective path validation approach requiring each SFC and NF to confirm the secure tag. The proposed validation model packets are encrypted using the hash technique, and the payload and a predetermined path validation code are used to create a certain title^23^. Because each packet must undergo a cryptographic operation as part of this hop-by-hop verification at each NF, there is a large processing burden on each NF.

Strong key management among all the NFs participating in the validation process is a crucial need for the secure implementation of the SFC validation procedure. We presumptively trusted the controller, Classifier, and NFs^24^. The Classifier is a logical entity implemented by adding batch hash computation and encryption capabilities to the router capability. The Classifier and NFs can support cryptographic operations^25^.

**Figure 5:**
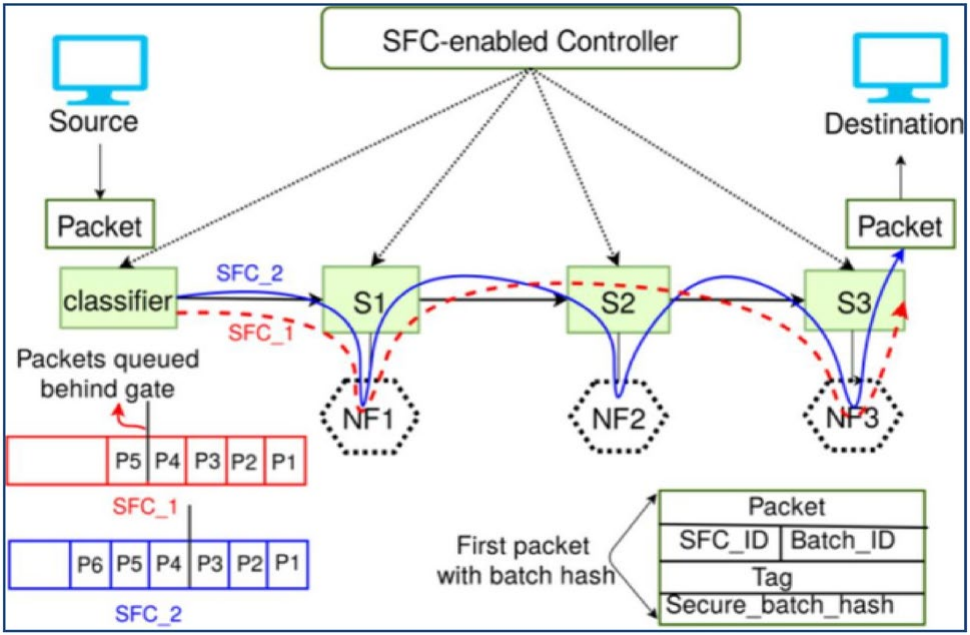
Working of the proposed EnsureS mechanism.

The controller uses a public–private key pair to communicate securely with NFs and classifiers. For each SFC, the controller creates a set of symmetric session keys and distributes them to the corresponding NFs, and classifiers have been mentioned in Fig. 5.

As an illustration, a controller might create a symmetric key and then share it with the Classifier and NFs by encrypting it with its private key. The key models of the proposed model are as follows.

### I. Lightweight service path validation with batch hashing

Two basic policies must be defined for batch hashing: a gated approach for creating batches and an encryption policy for making secure batches. The gated policy, which regulates how long a batching server must wait before closing the gate, is used to produce a batch. The server then processes each packet that has entered the gate one at a time^26^. The following are competing objectives of creating a batch.

- Processing as many as packets is feasible to minimize processing overhead and
- Attempting to wait for as little as possible to avoid increasing the average waiting time for the packet.

The following gated policies for a batching server are considered to assess a suitable compromise between these objectives.

**Figure 6:**
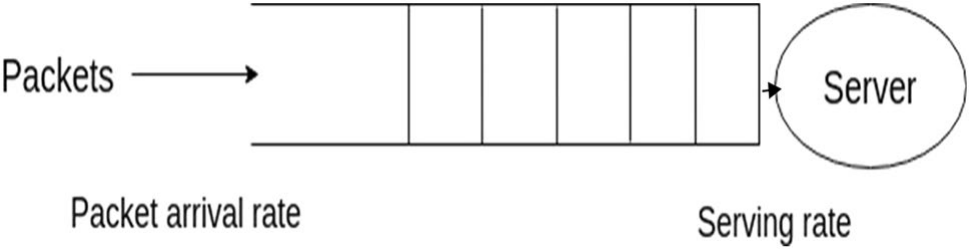
Packet processing at classifier in EnsureS mechanism.

Gated by number and time: under this policy, the batch server awaits the arrival of the Kth packet or the passing of a predetermined waiting time following the arrival of the first packet to the queue, whichever comes first. Packet forwarding is delayed because of the predetermined waiting time if batch hashing is used directly for service path validation, resulting in longer average waiting times between packets. The fixed waiting time must be accurately determined to adopt batch hashing efficiently^27^. Let the packets arrive in a queue at an arrival rate and inter-packet arrival time, as indicated in Fig. 6. The batch server processes packets at a rate independent of other packets or hash computation.

Nevertheless, the entire processing time of a batch of packets is represented as TP_total = TP_hash + TP_Encrypt, which accounts for both batch hash computation time and batch encryption time. The fixed hash's average packet size should be the maximum batch size if the T_Ptotal (pkt). The number of packets that arrive within the waiting period after the first packet arrives is the maximum batch size if TP_total^28^.

Encryption policy for batch hashing: the classifier must choose how to encrypt the batched packets after determining how many packets are served in a batch. Let K be the batch size or the number of packets in a batch determined by the GNT gated policy. Each NF of an SFC is required to encrypt the packet hash to verify the secure tag. The NF must perform a computationally intensive encryption operation for each packet. Concatenating the packets' hashes to lower computational costs^29^.

$${\text{i}}.{\text{e}}., {\text{BH }} = {\text{ H }}\left( {{\text{pkt1}}} \right) \, + {\text{ H }}\left( {{\text{pkt2}}} \right) \, + {\text{ H }}\left( {\text{P kt3}} \right) \, + \cdots \, + {\text{ H }}\left( {{\text{Pkt}}\_{\text{B}}} \right)$$

The batch hash BH is then encrypted to create SBH [BH] and is sent to NF_j_, Where 1 < j < n, n is the number of network functions in an SFC.

Secure tag generation with batch hashing: the packet hash and specified path validation code are used to create the secure tag, as shown below. The NFs can alter the packets in SFC; for example, NAT can change the source IP address and port number. As a result, the packet's hashes are divided into two categories: fixed hash and variable hash, except for the varying elements of the packet, such as the source IP address and source port, which can be the same for a specific SFC; the fixed hash is calculated for the entire packet, including the payload^30^.

1 $$\mathrm{Fixed }\_{\rm hash }=\mathrm{ Hash }(\mathrm{Pkt})$$

To prevent attacker-initiated packet alterations or packet tampering, variable hashes are produced for the mutable field, and RND is a random number.

2 $$\mathrm{Variable }\_{\rm hash }=\mathrm{ Hash }(\mathrm{mutable }\_\mathrm{fields }||\mathrm{ R N D })$$

Generation of PVC: the path validation code (PVC) values are created for each SFC. A special hash value called FlowID is designed for the SFC.

3 $$FlowID= MAC cpk (Path|| RND || Timestamp)$$

The computed FlowID is encrypted in the sequence of the NFs indicated in the SFC to produce the path validation codes. Let NF1 be SFC.

4 $${\mathrm{PVC}}_{1}={\mathrm{EK}}_{1}\left\{\left(\mathrm{FlowID}\right)\right\}$$

where k1 and k2 are the respective symmetric keys for NF1 and NF2, to verify the previous hop and upcoming hop in SFC, the controller sends a pair of PVC, designated PVC (i-1) and PVC I, to each NF participating in SFC.

5 $${\mathrm{PVC}}_{2}={\mathrm{EK}}_{2}\{{\mathrm{EK}}_{1}(\mathrm{FlowID})\}$$

### II. Workflow of EnsureS with batch hashing

The Classifier maintains a separate queue for each SFC and generates different batches per SFC since each NF in an SFC confirms the secure tag and hash at every hop^31^. Using Eqs. (1) and (2), the Classifier creates the fixed hash and variable hash for each packet in a batch.

**Figure 7:**
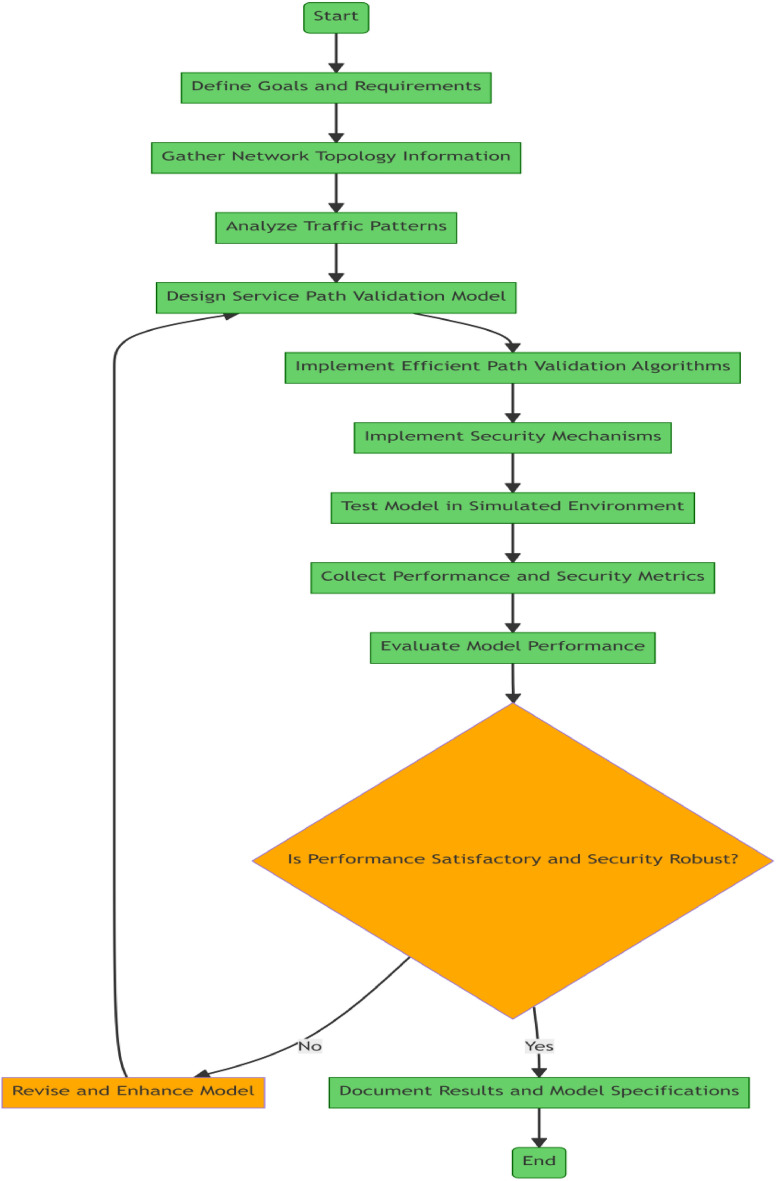
Workflow EnsureS model.

As shown in Fig. 7, the hash computation is continued until K packets are added to the queue or the first packet's waiting time has passed. If either condition is true, the Classifier computes the batch hash (BH) for K packets by concatenating their hashes and then encrypts it using the pre-shared symmetric key for SFC to create the secure batch hash.

The secure tag is calculated by executing an XOR on PVC, variable hash, and SBH (H(pkt)). Because BH and SBH [BH] are the same length, SBH (H (pkt)) is produced by reverse mapping of BHs H(pkt) to its H(pkt). The secure tag for the packet is formed as stated in Eq. after the secure batch hash has been computed at Eq. (6).

6 $${{\mathrm{Secure}}_{\rm{tag}}=\text{ PVCi XOR Esk }(\mathrm{fixed}\_{\rm hash}(\mathrm{pkt}))\text{ XOR variable}\_{\rm hash}(\mathrm{pkt}) }$$

With the first batch packet, the Classifier delivers the secure batch hash SBS [BH], SFC ID, and Batch ID. In a lossy network, SBH can be sent with the first three packets in case of packet loss. After receiving the batch's initial packet, each NF decrypts the SBH [BH] and stores the batch hash BH with the Batch ID and SFC ID. The NFs merely recomputed the packet hash H(pkt) and rapidly checked to see if H(pkt) could be found in BH when the batch's remaining packets were received. Suppose the security tag is recalculated by Eq. (7) and NF checks the path integrity. If H(pkt) is in the BH, a packet is forwarded, and the tag is updated and marked as "valid". NF discards the packet if either verification for it fails^28^.

7 $$\mathrm{SBH }[\mathrm{BH}] =\mathrm{ ESK }(\mathrm{BH})$$

## Results and discussion

The evaluation findings for EnsureS processing overhead gains when compared to current systems are provided in this section^32^.

### (A) Experimental setup and details

Ryu controller version 4.31 and Mini-net version 2.2.1 are used to implement the proof of concept. Four Open v Switches using Open Flow version 1.3 connected through a 1-GBPS link comprise a linear topology.

The classifier function is implemented by extending the first simple switch.VNFs with encryption and hashing capabilities are connected to switches. Processing overhead is determined by the time needed to compute validation data (including packet encryption and decryption) at each VNF for small-length SFC^33^. Table 2 presents a summary of the evaluation outcomes.

**Table 2:** Experimental results of proof of concept (PoC).

Methods	Values for 2 virtual network functions	Values for 3 virtual network functions
v Secure function chaining	156 µs	234 µs
c Secure function chaining	102 µs	154 µs
EnsureS (b-10) first packet	48 µs	72 µs
EnsureS (b-10) remaining	6 µs	s

### (B) Performance measuring

The following parameters were calculated for the proposed model and the existing model.

(i) Experimental setup and parameters: the necessary data for secure service path validation is produced by executing cryptographic operations while considering the packet's path. The processing overhead and latency caused by this service path validation affect packet forwarding. Using Python library functions, we replicated the cryptographic processes required for path validation for long-length SFC and compared the outcomes for vSFC, cSFC, and the proposed EnsureS scheme. In Table 3, the simulation parameters are listed^34^.

**table 3:** Simulation parameters.

Parameters	Values
Cipher suite	AES with CBC mode
Hash algorithm	SHA256
Packet arrival rate	Poisson distribution with mean 15 per ms
Packet size	1024, 512, 256 and 64 bytes
Number of VNFs in SFC (length of SFC)	2–12
Batch size	10–40
Timeout	(TP total/2)

Tables 4 and 5 present the quantity of hash, encrypted hash, and cryptographic operations (encryption or decryption) needed in the existing schemes vSFC, cSFC, and EnsureS with and without batches of 10. Hash-lib is used to create the hashes. Encryption uses the SHA-256 and AES functions in CBC mode. We assessed the packet forwarding time and additional processing overhead for vSFC, cSFC, and EnsureS. The comparative outcomes are provided in the subsections that follow. Here, Table 5 gives the cumulative cryptographic operations.

**Table 4:** Comparison of hash, encrypted hash, and cryptographic operations.

Method/types of operations	Encryption or decryption (AES)	Encrypted hash (HMAC)	Hash (SHA256)
vSFC	2*All NFs*P	0	2*all NFs*P
cSFC	2*complex NFs*P	2*all NFs*P	2*all NFs*P
EnsureS (w/0-b)	0	(all NFs + 1classifier)*P	2* (all NFs + 1 classifier)*P
EnsureS(b-k)	0	(All NFs + 1classifier)*P/k	2* (all NFs + 1 classifier)*P/k

**Table 5:** Comparison proposed model, when number of cryptographic functions required when all NFs = 3 (1 complex NF and two simple NF), no. of packets to process P = 100 in SFC validation methods.

Method/types of operations	Encryption or decryption (AES)	Encrypted hash (HMAC)	Hash (SHA256)
vSFC	2*3*100 = 600	0	2*3*100 = 600
cSFC	2*1*100 = 200	2*3*100 = 600	2*3*100 = 600
EnsureS (w/0-b)	0	(3 + 1)*100 = 400	2* (3 + 1)*2*100 = 800
EnsureS(b-k)	0	(3 + 1)*100/10 = 40	2*(3 + 1)*100/10 = 80

(ii) Extra processing overhead: processing overhead is the additional processing time needed to calculate the validation data. Figure 8 compares the extra processing overhead per packet as a function of the SFC's length (number of NFs). The packet payload is 64 bytes in size. To maintain the order of the NFs and prevent packet alteration in the vSFC example, each NF must complete the encryption and decryption operations on the full packet. As a result, vSFC has a high packet processing time that linearly rises with the number of NFs. In contrast to vSFC, the packet in cSFC is only partially decrypted by some NFs, such as IDS, for payload inspection, and other NFs create authentication fields through HMAC computation^35^.

**Figure 8:**
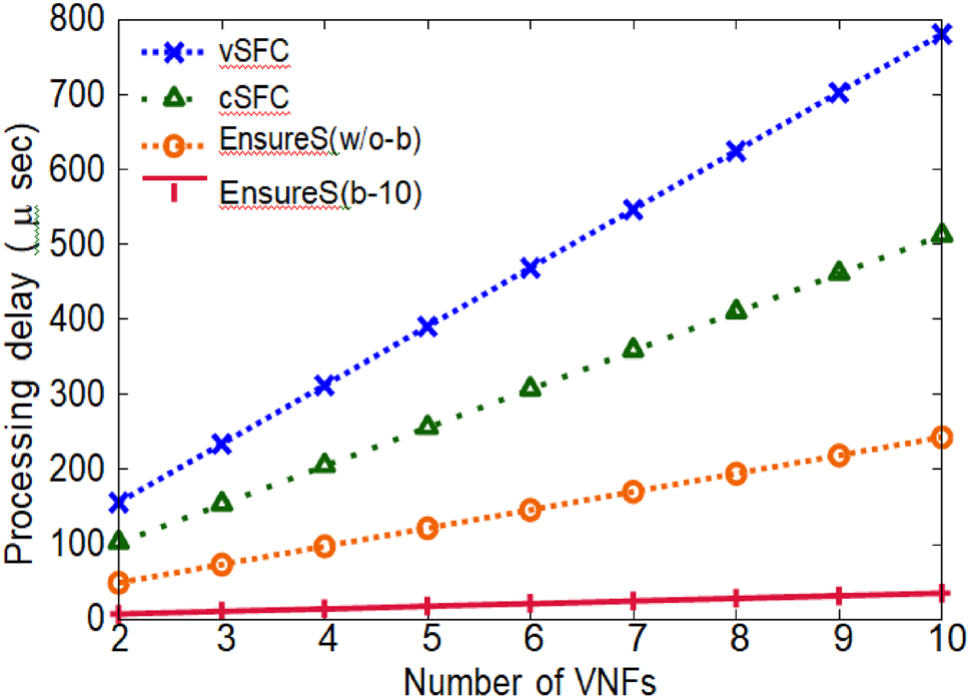
Comparison of extra processing overhead per packet (EnsureS vs existing scheme).

**Figure 9:**
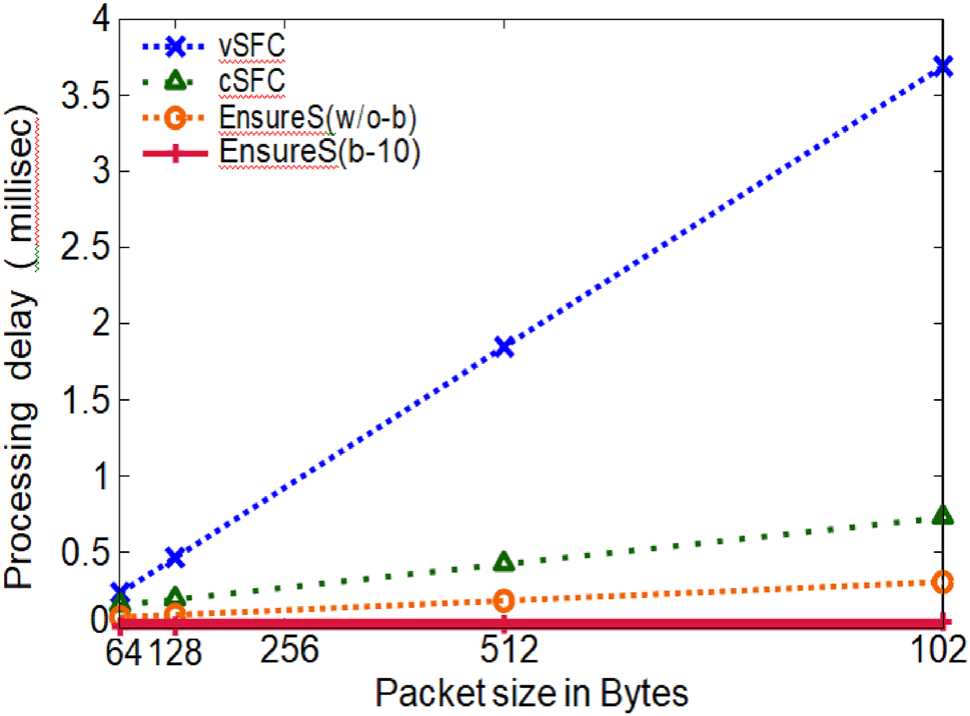
Comparison of packet processing latency per NF (EnsureS vs existing scheme).

If EnsureS verifies the SFC without a batch, it is denoted as EnsureS (w/o-b), which means it computes and delivers an encrypted hash for each packet that arrives without waiting for a collection to form. EnsureS (w/o-b) displays the least overhead compared to the current approach. Increasing the batch size K, representing the most packets considered in a batch, further reduces the processing overhead. K is regarded as 10 in Fig. 8. As a result, compared to vSFC and cSFC, the EnsureS scheme can minimize processing overhead per packet by 90% and 69%, respectively, due to batching^36^.

(iii) Packet processing latency: the complete packet is encrypted and decrypted at each NF in SFC for vSFC and cSFC. Figure 9 displays the latency added to packet processing at each NF due to cryptographic operations with varied packet sizes. Every NF in vSFC conducts encryption and decryption on the full packet, resulting in greater latency as packet sizes increase.

**Figure 10:**
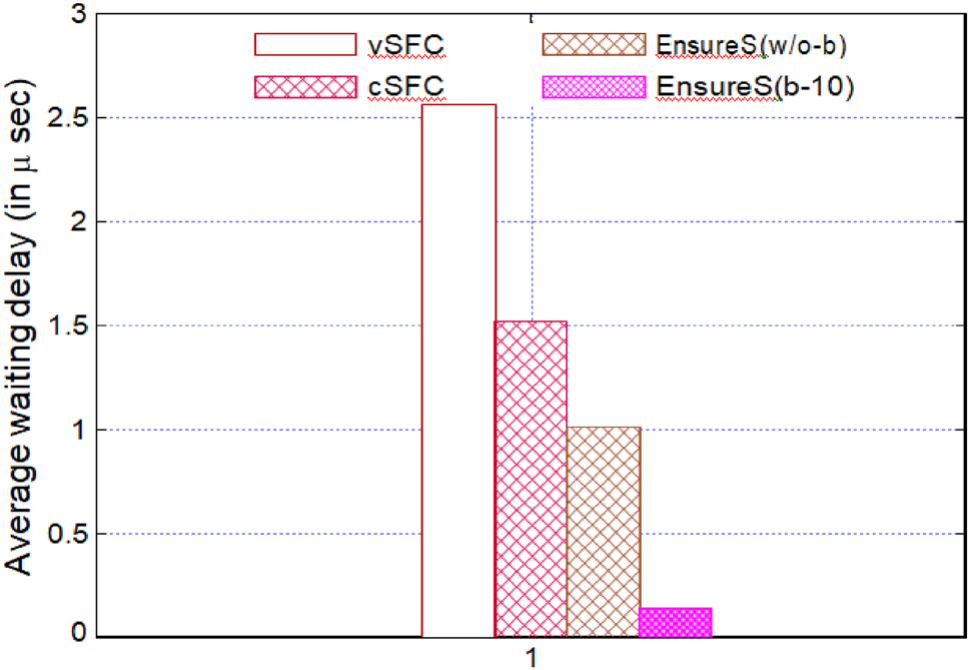
Average waiting time per packet in EnsureS with the existing scheme.

Compared to vSFC, the decryption procedure is skipped in cSFC if the NF is not changing the packet payload. The packet hash is calculated for EnsureS and used to create a batch hash. The batch hash for EnsureS (b-10) is 32 bytes, consistently significantly smaller than the original packet size of 64 bytes-1024.

(iv) Average waiting time: the average waiting time per packet calculated at the Classifier when using the validation process is shown in Fig. 10. Packets traverse a total of three VNFs' SFCs. The average waiting time for the system is calculated as 1/(−), where is the packet arrival rate, which follows the Poisson distribution with a mean of 15 packets per millisecond, and is the processing rate, which shows the number of packages processed each millisecond.

Because of the lengthy processing times for packet encryption and decryption at each NF, the average waiting time for vSFC is significant. Compared to vSFC, cSFC displays less waiting time because of encrypted hash computation and selective encryption. Unlike existing schemes, EnsureS with and without batching shows a shorter waiting delay per packet^37^. Figure 10 shows the average waiting time per packet in EnsureS with the existing scheme.

**Figure 11:**
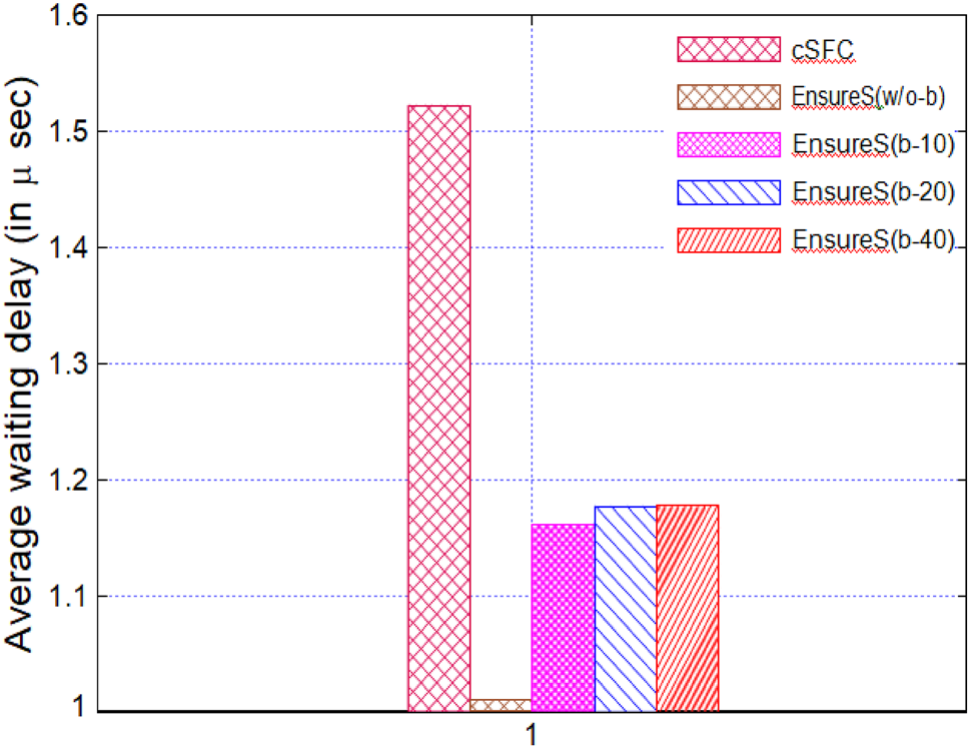
Average waiting time of the first packet in EnsureS with varying batch size.

The first packet of the batch must wait until the collection is formed because of the batching notion in EnsureS. As a result, we assessed the typical waiting time experienced for vSFC, cSFC, and EnsureS. The average waiting time for the first batch packet at the Classifier is shown in Fig. 11.

We noticed that the batching approach introduces a small waiting delay for the initial packet. The delay increases with batch size and stays constant because the Timeout is constant and equal to (T Ptotal/2). Thus, T Ptotal represents the total amount of time needed to compute a packet's hash and encrypt its hash. Compared to EnsureS without batching, the delay caused by batching is minimal, nearly 0.2 μs and substantially shorter than the waiting delay of cSFC. As a result, the batching-related delay in EnsureS is minimal and has little impact on packet forwarding^38^.

## Discussion

The findings from the experiments prove the effectiveness of the highly effective Protect Service Path Validation Models for SDN. The Proposed EnsureS Model exhibits remarkably better latency, throughput, and security effectiveness than the existing model. A latency comparison demonstrates that the Proposed EnsureS optimized the path validation procedure and substantially lowered data propagation delays, particularly in high network demand. Removing this latency is necessary for applications that operate in real-time, such as multiplayer gaming and video meetings.

Figures 8, 9, 10 and 11 presents the proposed and existing models' simulation results. The proposed model EnsureS achieved better outcomes for processing overhead, Packet processing latency and Average waiting time. It proves the efficiency of the proposed model. The experiment has shown that the batch size directly impacts the EnsureS performance. When there are more packets in a batch, EnsureS works better. The average delay time for EnsureS with and without batching at the Classifier is shown in Fig. 10. The average waiting time of EnsureS lowers as the batch size grows. Figure 11 demonstrates how the processing overhead in EnsureS can be greatly reduced compared to not using batching.

However, the greatest number of packets that can be considered in a batch is limited by the packet size and the SFC implementation strategy. Any header-based SFC implementation, like NSH, can create the EnsureS scheme because a metadata field can embed the batch hash. The maximum batch size for an SFC implementation setting using traditional Ethernet technology can be Ethernet MTU, the size of (first packet), which can be expanded further with a jumbo Ethernet frame to improve EnsureS performance.

The Proposed Model is suitable for contemporary data-intensive services because it can handle larger information volumes, as shown by the throughput assessment. The model's optimized path validation and effective utilization of resource strategies are responsible for this competitive advantage. The multi-layered safety features in the Proposed EnsureS Model offer improved defence against unauthorized access, theft of information, and cyber-attacks. The detection radar visualization effectively shows the model's all-encompassing safety measure, which protects the security and accuracy of communication over the network. The Proposed EnsureS Model is a crucial development in Software Defined Networks because of its effective path validation, increased throughput, and strong security. The experiment's results support its applicability and advantages in real-world network instances.

## Conclusion

A service path validation is crucial to overcome security risks in a communication network. The existing validation mechanisms protect against packet modification attacks and maintain path integrity, but they encounter a higher delay due to the demand for increased processing requirements per NF. To overcome these security issues, this research presented an efficient and secure service path validation model (EnsureS) in the software-defined network. The proposed EnsureS model is based on Lightweight service path validation with batch hashing and tag verification to combat security threats.

With new applications, there is an increase in demand for E2E services. Different network services with a particular quality of service requirements are frequently needed to deliver end-to-end services. Large-scale networks, including SD-WAN, are rapidly adopting SDN for the infrastructure's automated management, E2E quality assurance, and flexible placement of network operations. The logically centralized SDN architecture can facilitate E2E services for new applications. Yet, some difficulties and important problems must be resolved when deploying E2E services in an SDN context.

The proposed architecture allows a flexible and dynamic provisioning of resources and network operations. In experimental analysis, it is proven that compared to the existing Service path validation method, the proposed model avoids packet modification attacks significantly and lowers the processing overhead per packet.

The thorough findings, which examine system performance enhancements, demonstrate that the proposed SDN resolves the security challenges presented in this research.

In the future, we will build towards this research by employing Blockchain to further strengthen data security and privacy. Additionally, we plan to use this work in a larger ecosystem to analyze the SDN network operations. We will also implement the proposed model in a real-time environment and compare it with various robust techniques and security models.

## Data Availability

The dataset is available with the corresponding author based on individual requests.

## Abbreviations

SFC: Service function chaining
MD-SFC: Multi-domain service function chain
NFV: Network function virtualization
NF: Network functions
E2E: End-to-end
NFV: Network function virtualization
VNF: Virtual network function
SDN: Software-defined networking
EnsureS: Efficient and secure service path validation model
VMs: Virtual machines
SFC: Service function chaining
SPV: Service path validation
NFs: Network functions
IDS: Intrusion detection system
FW: Firewall
